# Quantitative fat-fraction analysis of the rotator cuff muscles on clinical sagittal and coronal T1-weighted MRI using deep learning algorithms

**DOI:** 10.1038/s41598-026-38108-3

**Published:** 2026-02-13

**Authors:** Hanspeter Hess, Alexandra Oswald, Keivan Daneshvar, Nicolas Gerber, Michael Schär, Matthias A. Zumstein, Kate Gerber

**Affiliations:** 1 Department of Orthopaedic Surgery and Traumatology, Inselspital, Bern University Hospital, University of Bern, Bern, Switzerland; 2 Department of Diagnostic, Interventional and Paediatric Radiology (DIPR), Bern University Hospital, Inselspital, University of Bern, Bern, Switzerland; 3 Shoulder, Elbow & Orthopaedic Sports Medicine, Orthopaedics Sonnenhof, Bern, Switzerland; 4 Faculty of Medicine, University of Bern, Bern, Switzerland; 5 Faculty of Medicine, Health and Human Sciences, Macquarie University, Sydney, Australia

**Keywords:** Anatomy, Computational biology and bioinformatics, Diseases, Health care, Medical research

## Abstract

Increased fatty infiltration of the rotator cuff muscles is a primary prognostic factor for poor surgical outcomes of rotator cuff repair surgery. Preoperative fat assessment currently relies on the qualitative Goutallier classification using magnetic resonance imaging (MRI). This method suffers from high observer variability and only assesses a single slice. The aim of this study was to use deep learning to predict quantitative, voxel-wise fat fraction (FF) from standard T1-weighted MRIs. A deep learning-based algorithm was developed for automatic FF prediction using a voxel-wise, five-class system. The network was trained on 75 patients using paired T1-weighted and 2-point Dixon MRI, with rotator cuff muscles segmented in coronal and sagittal planes. It was validated on 24 patients. The proposed algorithm was significantly more accurate than a binary fat classification approach (*p* < 0.001). Average whole muscle FF calculation errors (mean ± standard deviation) ranged from − 0.5 ± 2.2% to 2.3 ± 3.9% compared to Dixon MRI measures. Deep learning enabled an accurate, voxel-wise FF quantification using clinical T1-weighted MRIs. This method allows for muscle FF distribution analysis, providing a more comprehensive assessment, that can improve prognosis analysis and optimise treatment planning.

## Introduction

Rotator cuff muscle fatty infiltration is a primary risk factor of structural repair failure following surgical rotator cuff tear (RCT) repair^1–5^. The decrease in muscle tension and contractility associated with rotator cuff tears leads to a loss of muscle tissue and an increase in adipose tissue within the epimysium, resulting in reduced muscle strength, reduced muscle quality^6^, reduced force per cross-sectional area^7^ and reduced extensibility^8^; characteristics important for the sufficiency of RCT repair. Goutallier et al. described a qualitative visual categorization of fat infiltration (1: no fat, 2: few fatty streaks, 3: less fat than muscle, 4: same amount of fat and muscle, 5: more fat than muscle) on computed tomography (CT), correlating higher fatty infiltration to poor surgical outcome^9^. Fuchs et al. adapted this method to analyse fatty infiltration on oblique sagittal T1-weighted magnetic resonance imaging (MRI). Analysis was performed on a single MRI slice, known as the Y-view, defined as the most lateral sagittal slice on which the scapular spine merges with the scapular body^5^. To perform this classification on T1-weighted MRI, hyperintense areas within the muscle tissue have conventionally been interpreted as fatty infiltration. Despite large intra- and inter-observer agreement, this subjective metric remains a standard for assessing RCT repair suitability^10,11^. Due to the large intra- and inter-observer variability, simplified classifications of only three classes have been proposed^5,12,13^, however, five class categorisation remains standard for RCT prognosis analysis.

While fatty infiltration remains one of the most relied upon metrics for RCT treatment planning, Vidt et al. showed that current clinical scores of fatty infiltration assessed on a single 2D slice are not representative of 3D measurements^14^. In RCT patients with significant tendon retraction, the muscle’s position is retracted relative to the Y-slice^15^ which additionally creates a variable region of interest for the fat fraction analysis from patient to patient^16^.

To address these limitations, Werthel et al. presented methods for whole-muscle fat fraction analysis in CT^17^, relying on manual segmentation of the rotator cuff (RC) muscles, and intensity thresholds to separate fat and muscle tissue^18^. Riem et al. more recently introduced an approach for 3D fat fraction analysis on standard clinical sagittal T1-weighted MRI using deep learning^19^. They trained a convolutional neural network to segment the RC muscles and fat and muscle separation. However, as their method classified the tissue in each voxel as either fat or muscle, the voxel-wise percentage of fat within the tissue was not quantified. Their reliance on sagittal imaging also excluded approximately 60% of the RC muscle volume, which is outside of the image field of view (FOV) in 91% of T1-weighted images obtained during typical clinical workflow^20^.

While T1-weighted MRI is commonly used in RCT diagnosis, it provides only a binary classification of fat and muscle tissue within the rotator cuff muscles. Alternatively, MRI techniques such as the Dixon method^21^ provide fat suppression imaging that enables voxel-wise fat fraction quantification of the rotator cuff muscles at the Y-slice^22–24^ or of the whole muscle^25–27^. These techniques have enabled 3D quantitative analysis of intramuscular fat distribution, revealing heterogeneity across individual rotator cuff muscles and different pathologies^28–30^. It has also been shown that torn muscles not only have higher fatty infiltration but also increased intracellular lipid^6^, which cannot be spatially resolved, and may be missed through intensity thresholding of a T1-weighted MRI^31,32^. Moreover, volumetric quantitative fat fraction assessment has been shown to correlate with physical characteristics of the musculotendinous unit, such as extensibility and stiffness^8^, characteristics important to RCT repair success.

While Dixon MRI has shown promising results for RCR prognosis analysis, it is generally not acquired as part of a standard MRI study of the shoulder at most centres. We propose that deep learning could alternatively be used to obtain an accurate 3D fat fraction measurement of the rotator cuff muscles from standard diagnostic T1-weighted MRI data, by training a convolutional neural network to predict the voxel-wise fat content based on corresponding Dixon MRI. Herein, we present an evaluation of the method relative to state-of-the-art analysis on Dixon MRI and highlight the effect of binary fat classification on fat fraction quantification. To address the limited FOV of the standard sagittal T1-weighted MRI, and to enable quantitative fat fraction analysis in a larger FOV, we trained our prediction networks on both sagittal and coronal T1-weighted MRIs.

## Methods

### Data

MRI data was retrospectively collected with approval of the ethical committee of the canton of Bern, Switzerland (no. 2021 − 00326), and the study was conducted in accordance with the Declaration of Helsinki. 99 patients who presented at the Inselspital, University Hospital of Bern, Switzerland (*N* = 69) and Orthopaedics Sonnenhof, Bern, Switzerland (*N* = 30) with partial- or full-thickness RCTs between 2019 and 2023 were retrospectively enrolled in this study. General consent was obtained from all patients. Inclusion criteria included the previous acquisition of diagnostic sagittal and coronal T1-weighted MRI (without fat suppression) and transversal 2-point Dixon MRI (2pDixon) with a FOV that included the entire shoulder, and age greater than 18 years. All MRIs were acquired on a Siemens 3.0 Tesla scanner equipped with a dedicated shoulder coil. T1-weighted MRI scans in the coronal plane had an in-plane resolution of 0.31–0.39 mm, and a slice-thickness of 3.3–3.6 mm, and in the oblique sagittal plane, an in-plane resolution of 0.31 mm and a slice-thickness of 3.5–3.7 mm. The isotropic 2pDixon MRIs were acquired in the transversal direction (resolution: 1.1–1.2 mm).

The Goutallier grades (GGs) of all rotator cuff muscles were evaluated by two experts individually on sagittal T1-weighted MRI according to the methods proposed by Fuchs et al.^5^. Cases of non-agreement were re-evaluated by the experts, and a consensus was reached.

### Quantitative fat fraction

**Fig. 1:**
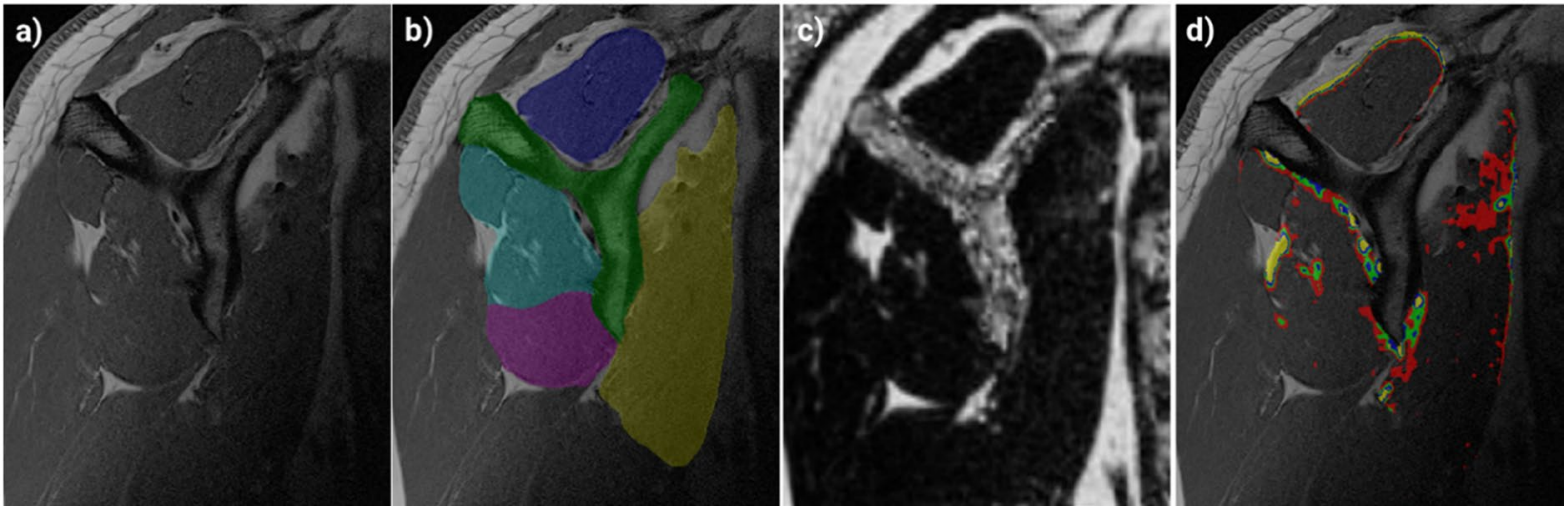
Sagittal MRI of a shoulder at Y-slice. **a**) T1-weighted MRI; **b**) automatic segmentation of the scapula (green), supraspinatus muscles (dark blue), infraspinatus muscle((cyan), teres minor muscle (magenta), subscapular muscle (yellow); **c**) quantitative muscle fat fraction volume, transferred from 2pDixon MRI; **d**) fat fraction classes inside the rotator cuff muscles, no color: <15% fat, red: 15–30% fat, green 30–45% fat, blue: 45%- 60% fat, yellow: > 60% fat.

**Fig. 2:**
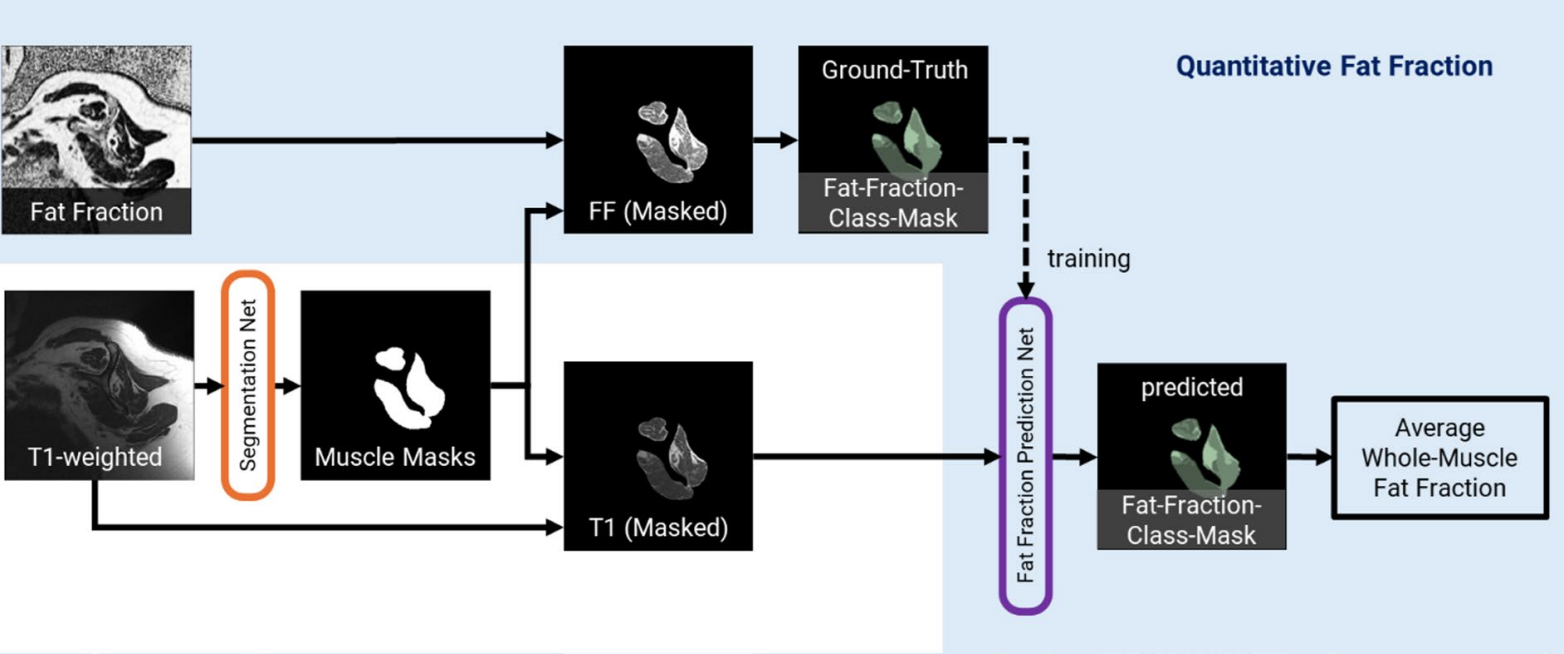
Workflow for voxel-wise quantitative fat fraction prediction using a deep learning algorithm. T1-weighted MRIs are processed through a segmentation network to generate masks for the humerus, scapula, and the four rotator cuff muscles. These masks are used to isolate muscle regions in the aligned T1-weighted and fat-fraction images. Within each muscle in the fat fraction images, five classes with increasing ratios of fat to muscle tissue are defined. These ground-truth fat fraction class masks guide the training of a quantitative fat fraction prediction network on the T1-weighted MRI, resulting in predicted fat-fraction class masks.

The four rotator cuff muscles, the supraspinatus (SSP), infraspinatus (ISP), subscapularis (SSC), and teres minor (TM), along with the humerus and scapula, were automatically segmented into different labels in the T1-weighted MRIs (Fig. 1) using our previously validated deep learning-based algorithm^33^. Segmentations were verified by an expert and manually corrected if required. The voxel-wise quantitative fat fraction for each shoulder was obtained from the fat-fraction image of the 2pDixon MRI. T1-weighted MRI and the in-phase 2pDixon MRI were registered and the voxel-wise fat-fraction values from 2pDixon MRI were transferred to the coronal and the sagittal T1-weighted MRI of the same shoulder. For registration the “normalized mutual information” metrics^34^ with “Adaptive Stochastic Gradient Decent” Optimizer was used using a Python (v3.10) algorithm with SimpleITK (v2.0.0), ITK (v5.3.0), and SimpleElastix (v0.10.0) libraries. All registration results were checked manually using synchronized viewers of the fixed and registered image. On these quantitative muscle fat fraction volumes as well as the T1-weighted MRI, information outside of the muscles was removed by setting all voxels outside the segmented RC-muscle mask regions to zero (Fig. 2). The average whole-volume fat fraction of each muscle was calculated as the mean fat fraction of all voxels within each muscle volume.

#### Voxel-wise fat fraction prediction network

Fat content of each voxel of the quantitative muscle fat fraction volumes were categorized into five classes: class 0 for voxels containing less than 15% fat, class 1 for voxels with between 15% and 30% fat, class 2 for voxels with between 30% and 45%, class 3 from 45% to 60%, and class 4 over 60% fat to produce fat-fraction-class-masks (Fig. 1).

A deep learning network was trained to predict the voxel-wise fat fraction categories of the rotator cuff muscles from sagittal and coronal T1-weighted MRI (Fig. 2). The MRI data was randomly split into a training dataset (75 shoulders: 75 coronal-, 75 sagittal-oriented MRI) and a test dataset (24 shoulders: 24 coronal-, 24 sagittal-oriented MRI).

The training dataset was randomly split into five folds, with all MRIs from the same patient kept within the same fold. A 3D full-resolution U-Net was then trained on the training dataset for 250 epochs using the nnU-Net framework^35^ in a five-fold cross-validation process. The standard nnUNetTrainerV2 with all standard fixed parameters were used for training. The rule-based parameters for our dataset (median image size [voxels] of 22.0, 394.5, 351.5 and spacing [mm] of 3.54, 0.31, 0.31) and the used GPU (NVIDIA GeForce RTX 3090) were: Input patch size [voxel] of 256, 320, 20, a batch size of 2 and the z-score normalization Method. The ensemble of the outputs of the five trained networks was used as the final predicted mask.

#### Accuracy evaluation

The accuracy of the trained fat fraction prediction network was evaluated on the test dataset. The network was applied to each of the masked T1-weighted MRIs of the test dataset (24 coronal, 24 sagittal). The average volumetric fat fraction of each RC muscle was quantified using the muscle-mean of the quantitative fat fraction values derived from the predicted fat-fraction-class-masks.

The accuracy of the proposed method was also tested against a more traditional binary fat segmentation method, usually used to evaluate fat fraction on CT^17^ and on T1-weighted MRI^19^. While the results of a binary segmentation fat fraction measure can vary greatly depending on the applied segmentation method, we reduced the influence of segmentation error by utilising the corresponding quantitative muscle fat fraction volumes from the Dixon MRI. A binary threshold on these quantitative values was applied (> 40% fat fraction assigned to fat, to approximate the visual appraisal of bright tissue on T1-weighted). To calculate the average whole muscle fat fraction from the resulting binary mask, the ratio of voxels inside the fat-mask to the total number of voxels within the muscle-mask was calculated. In this approach, “fat-voxels” were considered as 100% fat while “muscle-voxels” were considered as 0% fat.

The average whole-muscle fat fractions obtained using our proposed method, as well as by the traditional binary fat segmentation method, were compared to those calculated from the ground-truth quantitative muscle fat fraction volumes. Significance of differences in absolute errors of the two methods compared to the ground-truth was tested using the Wilcoxon signed-rank test, calculated in Python using the SciPy (v1.15.2) library.

#### Fat fraction distribution

For analysis of the fat fraction distribution of the RC muscles along the frontal axis, the mean fat fraction within each muscle mask was calculated for each sagittal slice. Only slices in which the mask area of the specific RC muscle was larger than 50 mm^2^ were included. The Y-slice^5^ was detected automatically as the most lateral sagittal slice where the scapular spine shows simultaneous contact with the glenoid, coracoid, and acromion processes. This was achieved using a Python script first detecting lateral and medial direction by comparing the center of the humeral (lateral) and the scapula (medial) mask and then iterating through the sagittal slices from lateral to medial, identifying the first slice where the scapula mask formed one single, connected component. Y-slice detection was checked manually.

The lateral distance of each slice to the Y-slice was calculated and resampled to positions with five-millimeter steps. To enable a comparative analysis on the coronal MRI, the fat fraction and muscle masks were interpolated to the resolution of the sagittal MRI of the same patient. Mean and standard deviation of the slice-wise fat fraction were determined relative to the lateral distance to the Y-slice.

**Table 1:** Goutallier grade of the four rotator cuff muscles for the whole cohort.

	Goutallier grade				
	0	1	2	3	4
Supraspinatus	18	38	32	7	4
Infraspintaus	11	53	33	0	2
Teres Minor	53	37	8	0	1
Subscapularis	34	47	13	1	4

## Results

Of the muscles analysed in this study, 116 had GG 0, 175 had GG 1, 86 had GG 2, 8 had GG 3, and 11 had GG 4 (Table 1). The quantitative fat fractions (mean ± standard deviation) at the Y-slice were measured as 15.3 ± 9.2% (SSP), 13.7 ± 9.0% (ISP), 11.6 ± 6.7% (TM), and 15.7 ± 9.7% (SSC), and the whole muscle fat fractions as 15.4 ± 8.5% (SSP), 13.5 ± 7.2% (ISP), 11.8 ± 4.7% (TM), and 15.1 ± 8.3% (SSC) (Table 3).

The quantitative fat fraction (mean ± standard deviation) calculated at the Y-slice of the muscles for each GG class is as follows: GG 0: 9.9 ± 3.3%; GG 1: 12.7 ± 4.4%; GG 2: 17.3 ± 6.7%; GG 3: 22.0 ± 6.5%, and GG 4: 48.8 ± 22.0%.

### Voxel-wise fat fraction prediction network accuracy

Using our deep learning method, the average whole-muscle fat fraction of the test cohort was predicted with accuracies (mean ± standard deviation) of −0.5 ± 2.2% (SSC), 1.3 ± 1.9% (ISP), 2.3 ± 3.9% (SSP), 1.5 ± 2.8% (TM) in the sagittal plane, and 0.1 ± 2.3% (SSC), 1.4 ± 2.0% (ISP), 1.7 ± 2.1% (SSP), 0.4 ± 3.5% (TM) in the coronal plane, as compared to the ground-truth fat-fraction-class-masks. The largest mean error of 2.3% was observed in the calculation of the fat fraction of the SSP in the sagittal MRI.

**Table 2:** Mean and standard deviation of the ground truth and predicted quantitative fat fractions in the sagittal and coronal planes of the RC muscles over the whole muscle.

	ground-truth quantitative fat [%]				automatic quantitative fat (sagittal) [%]				automatic quantitative fat (coronal) [%]			
	sagittal		coronal		measurements		errors		measurements		errors	
	mean	SD	mean	SD	mean	SD	mean	SD	mean	SD	mean	SD
Subscapularis	14.8	± 7.9	15.5	± 7.6	14.2	± 6.8	−0.5	± 2.2	15.6	± 7.9	0.1	± 2.3
Infraspinatus	12.1	± 4.4	14.2	± 4.7	15.5	± 4.7	1.3	± 1.9	13.5	± 4.0	1.4	± 2.0
Supraspinatus	15.2	± 6.1	15.4	± 6.6	17.8	± 8.2	2.3	± 3.9	16.9	± 6.4	1.7	± 2.1
Teres Minor	11.8	± 5.1	12.4	± 4.7	14.0	± 4.4	1.5	± 2.8	12.2	± 3.2	0.4	± 3.5

The whole muscle average fat fraction measurement accuracy of our proposed deep learning based voxel wise fat fraction method were significantly higher (Wilcoxon signed-rank test, *p* < 0.001) than those obtained using a traditional binary fat/muscle classification approach on the same data, even when assuming a perfect separation based on the ground truth labels: −6.5 ± 4.0% (SSC), −5.9 ± 1.5 (ISP), −5.7 ± 2.5% (SSP) and − 6.6 ± 1.1% (TM) (Table 3).

**Table 3:** Comparison of the fat fraction classification systems on the test dataset (coronal and sagittal). Left: mean and standard deviation of the mean quantitative fat fraction of the RC muscles. Middle: fat fraction of the RC muscles with binary fat/muscle separation. Right: proposed fat-fraction-class-masks method for quantitative fat fraction measurement.

	quantitative fat (GT) [%]		quantitative fat (fat mask) [%]				automatic quantitative fat (proposed method) [%]			
	measurements		measurements		errors		measurements		errors	
	mean	SD	mean	SD	mean	SD	mean	SD	mean	SD
Subscapularis	15.2	± 7.7	8.7	± 11.3	−6.5	± 4.0	14.9	± 7.3	−0.2	± 2.2
Infraspintaus	13.1	± 4.6	7.3	± 5.6	−5.9	± 1.5	14.5	± 4.4	1.4	± 1.9
Supraspinatus	15.3	± 6.3	9.6	± 8.5	−5.7	± 2.5	17.3	± 7.3	2.0	± 3.1
Teres Minor	12.1	± 4.8	5.6	± 5.0	−6.6	± 1.1	13.1	± 3.9	1.0	± 3.2

### Fat fraction distribution

**Fig. 3:**
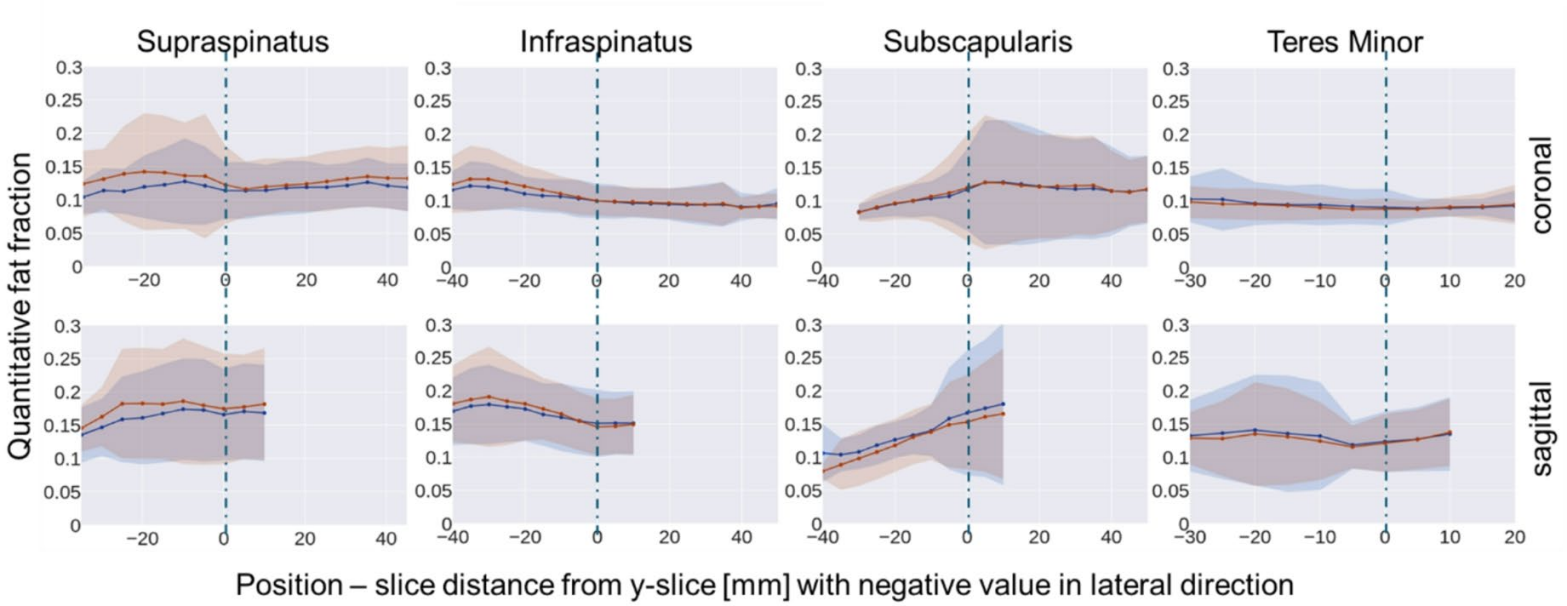
Ground truth (blue) and predicted (red) slice-wise quantitative fat fraction of the four RC muscles of the test dataset (Mean and standard deviation). Top row: Analysis on coronal T1-weighted MRI; Bottom row: Analysis on sagittal T1-weighted MRI.

**Fig. 4:**
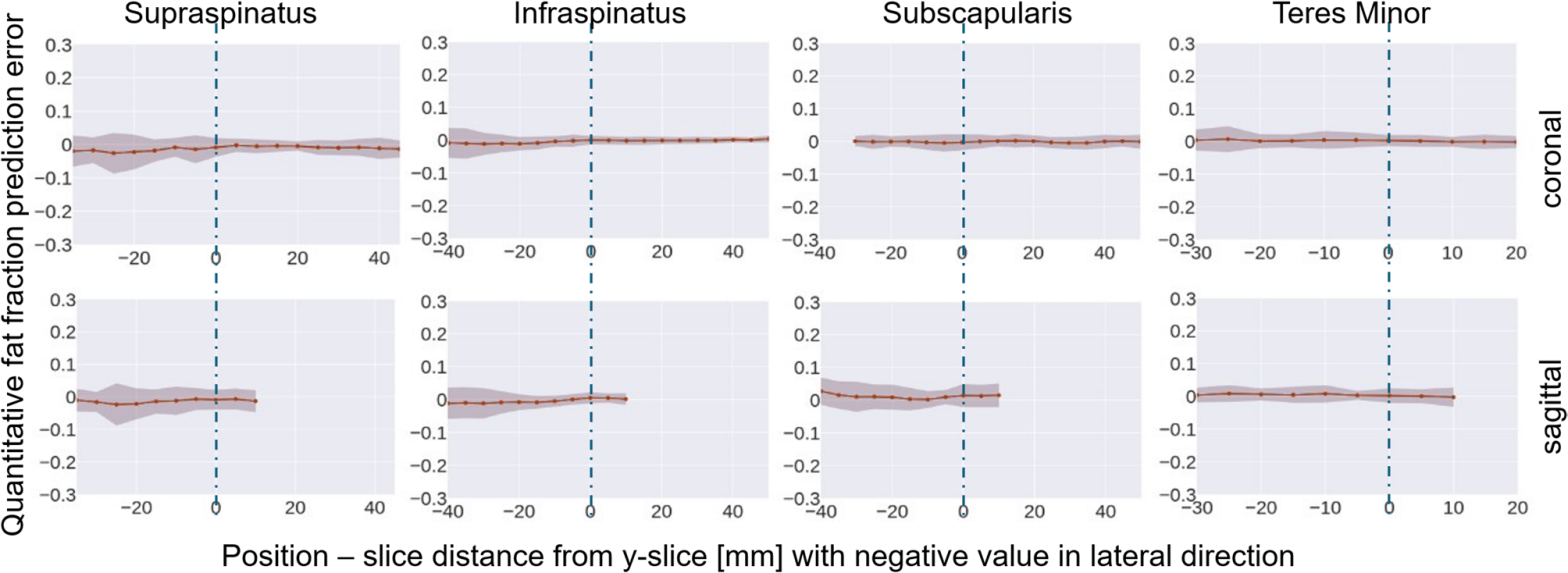
Mean and standard deviation of the errors of the predicted slice-wise quantitative fat fraction error of the four RC muscles of the test dataset compared to the corresponding ground truth. Top row: Analysis on coronal T1-weighted MRI; Bottom row: Analysis on sagittal T1-weighted MRI.

The Dixon fat fraction (blue) and deep learning predicted (red) slice-wise fat fraction (mean and standard deviation) along the frontal axis of the test dataset is depicted in Fig. 3 relative to the lateral distance to the Y-slice, for each RC muscle. The error of the predicted slice-wise fat fraction (mean and standard deviation) compared to the ground truth value in the same slice for each RC muscle is depicted in Fig. 4.

Slice-wise fat fraction and error were only calculated for slices where information of 20 or more out of the 24 test patients of the specific muscle was available.

## Discussion

In this study, we introduced a novel method for fully automatic quantitative fat fraction measurement of the rotator cuff muscles on clinical sagittal and coronal T1-weighted MRI. Training the deep learning algorithms on chemical-shift-based MRIs allowed for an accurate quantitative assessment of fat fraction. The network was trained on both sagittal and coronal MRIs, allowing for the analysis on an increased FOV and a more comprehensive analysis of the fat content within the RC muscles.

In our cohort, a majority of muscles were classified as GG 1. The TM was predominantly graded as GG 0, while the SSP also had a high proportion of GG 2 and 3, and 4.8% of the muscles were classified as GG 3 or 4. We have observed a large overlay of the quantitative fat fraction of muscles rated as GG 1 and 2, as well as 2 and 3, which aligns with the findings of Nardo et al.^22^.

**Fig. 5:**
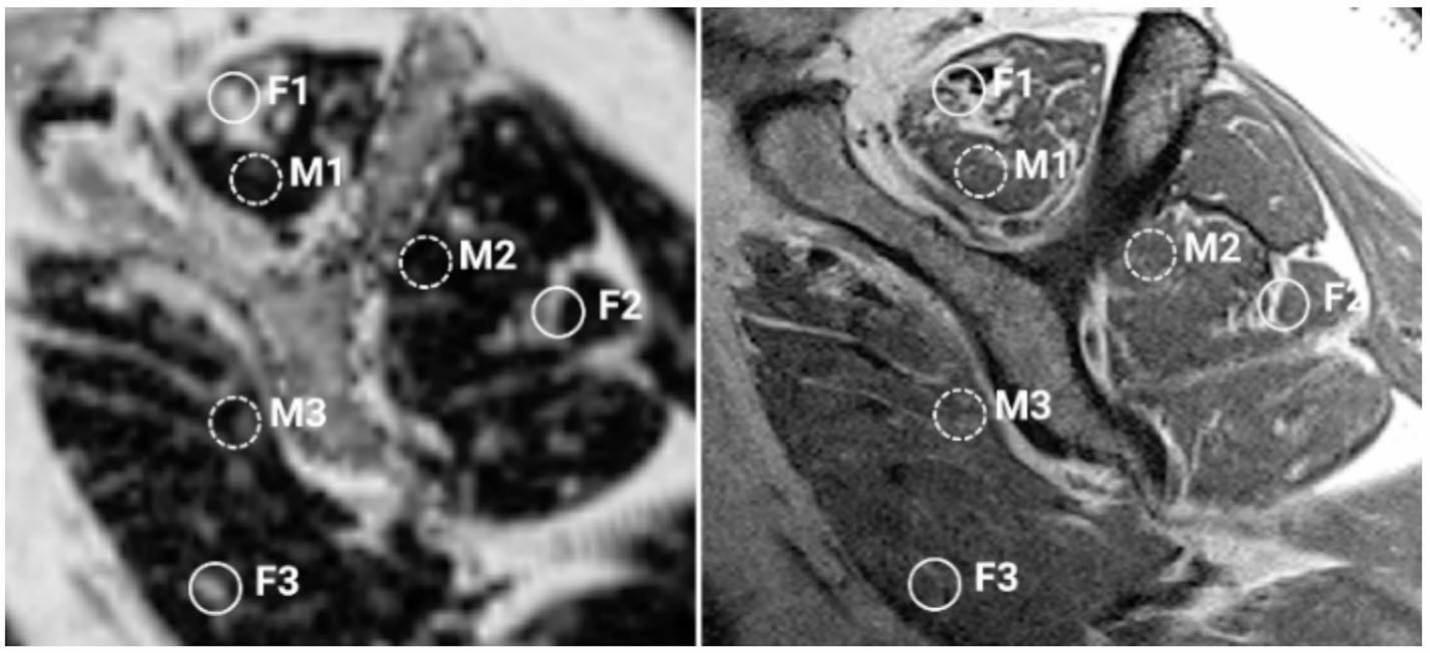
sagittal MRI from a patient with increased fat fraction of the RC muscles. Left: fat fraction image, right: T1-weighted MRI. Comparison of appearance in T1-weighted MRI and fat fraction image at the same location: all fatty streaks have similar fat fraction values; however, the T1-value differs: F1) T1: 291, F2) T1: 233 (80% of T1 of F1), F3) T1: 87 (30% of T1 of F1). Muscle tissues have similar values in T1-weighted MRI; however, they contain different amounts of fat fraction: M1) FF: 9% M2) FF: 10%/M3) FF: 30%.

In our cohort, binary fat classification using quantitative fat-fraction thresholding resulted in a substantial underestimation of the whole muscle fat fraction. All binary classification methods, including state-of-the-art deep learning-based segmentation methods, define the tissue in each voxel as either 100% muscle (0% fat) or 100% fat and thus exclude information regarding the percentage of fat in the tissue. For example, some muscle tissue in the SSP (Point M1 in Fig. 5) contains 9% fat, while some in the SSC (Point M3 in Fig. 5) contains 30% fat; however, a binary classification would define both as 0% fat. These differences in fat content within muscles and fat tissue are entirely neglected by all binary fat and muscle separation approaches, irrespective of the method employed for the binary classification. With our chosen threshold of 40% fat-fraction, designed to replicate the visual binary classification of fat and muscle on T1-weighted images, this approach leads to an approximate 50% underestimation of the total fat fraction compared to full quantitative DIXON data. While lowering this threshold (e.g., to 30% or 35%) would mathematically bridge this gap and yield higher overall fat estimates, it would introduce significant over-segmentation by including voxels that do not anatomically correspond to the macro-structural fatty streaks observed clinically. While the absolute fat volume is sensitive to the chosen threshold, the primary goal of this study was to benchmark our proposed multi-class method against the binary nature of current state-of-the-art manual segmentation.

**Fig. 6:**
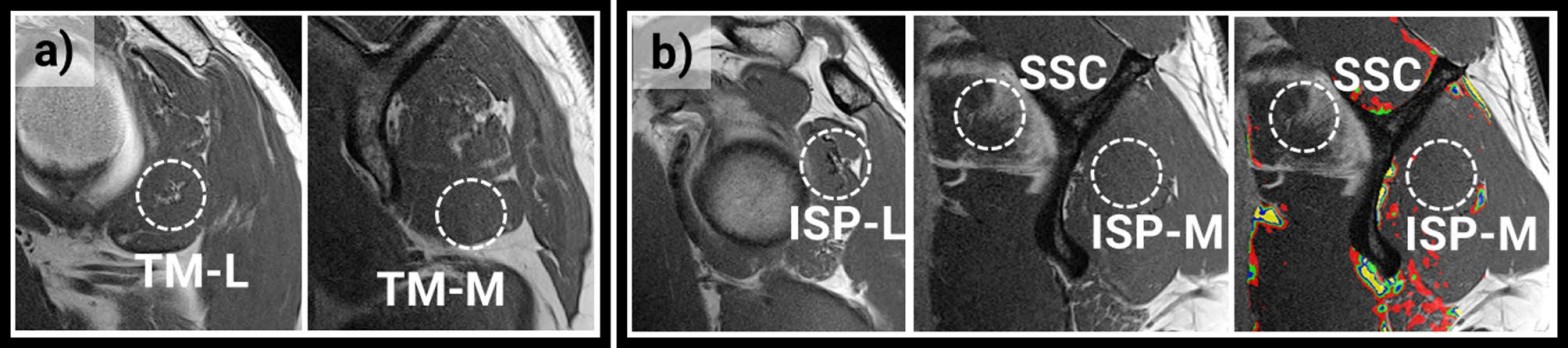
**a**) sagittal MRI of a Teres minor muscle with fatty infiltration in the lateral part (TM-L) and no visible fatty infiltration at the Y-slice (TM-M). **b**) sagittal MRI of an Infraspinatus muscle with fatty infiltration around the tendon in the lateral part (ISP-L) and barely any visible fat in the medial part (ISP-M). Contrast agent erroneously injected into the Subscapularis muscle and fat is hard to differentiate because of the similar intensities (SSC) is however successfully recognized by the trained deep learning algorithm which predicts a low fat fraction at this location.

The decreasing contrast and signal-to-noise ratio in T1-weighted MRI scans further from the coil hinders accurate manual labelling of fat across the entire image. Figure 5 displays a sagittal MRI with different locations of high fat fraction. The intensity at the high-fat location in the SSC (Point F3 in Fig. 5) is only 30% of that at the high-fat location in the SSP (Point F1 in Fig. 5). Moreover, the presence of erroneously injected contrast agent in the muscle complicates fat fraction evaluation in T1-weighted MRIs, because contrast agent is difficult to distinguish from fat due to their similar intensities. The proposed deep learning approach, trained on transferred quantitative fat fraction volumes from 2pDixon, overcomes these issues as the network learns, for example, to distinguish fat from contrast agent (SSC in Fig. 6).

The errors of the automatic fat fraction analysis using the predicted fat-fraction-class-masks were considerably lower compared to those introduced by the binary fat classification approach. Transferring voxel-wise quantitative fat fraction from the 2pDixon MRI enabled the analysis of fat content in coronal T1-weighted MRI, despite muscle fibres and fat streaks running perpendicularly to the high-resolution axis of the MRI and are therefore not distinctively visualized. Furthermore, the predicted slice-wise quantitative fat fraction deviated minimally from the ground truth across the entire length of the muscles (Figs. 3 and 4). Compared to sagittal MRI, coronal MRI allowed for fat fraction analysis in a larger medial FOV (Figs. 3 and 4).

**Fig. 7:**
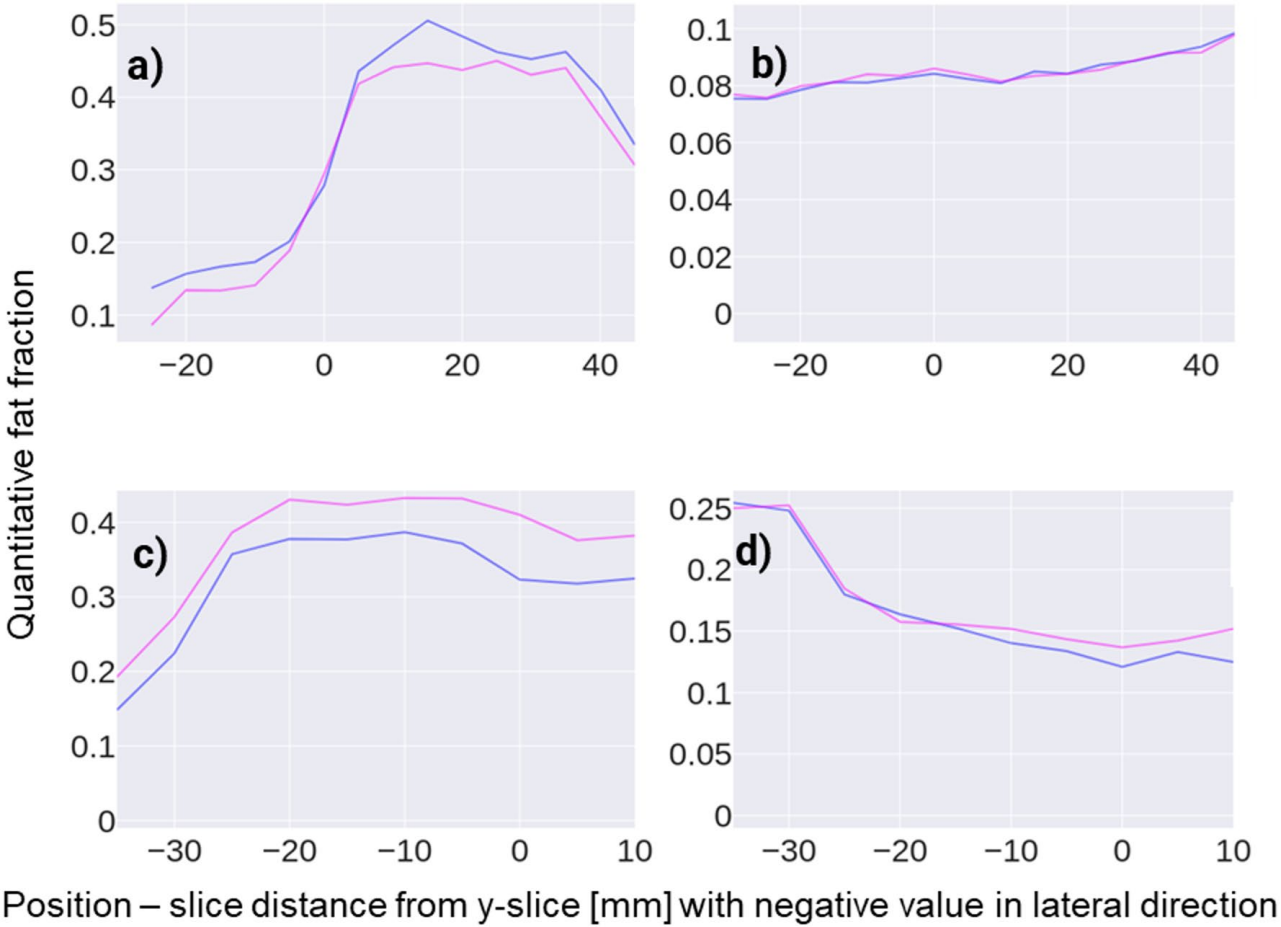
Predicted (magenta) and ground-truth (blue) quantitative fat fraction distribution of individual muscles: subscapularis muscle in coronal MRI with increased fat fraction medial to the Y-slice **a**) and subscapularis muscle in coronal MRI with constant fat fraction; **b**) supraspinatus muscle in sagittal MRI with decreased **c**) and increased fat fraction in the lateral segment.

In contrast to Santago and Seitz, who divided the muscles into five and three parts, respectively^28,29^, we analysed the muscles in 5-millimetre increments along the muscle direction. In our cohort, no overall change in mean fat fraction along the muscle was observed. However, considerable variation in fat fraction along the muscle was observed in individual cases (Figs. 6 and 7). The low prediction error of the full-muscle quantitative fat fraction shows that the proposed method allows for accurate fat fraction distribution analysis on large cohorts without the need for special MRI sequences.

In the future, we will use this method to investigate how the distribution of fat within muscles influences: (1) the clinical condition of the patient, (2) the outcomes of rotator cuff repair surgeries, and (3) whether increased fat fraction around the tendon is a different predictive parameter compared to increased fat fraction in the muscle belly. Additionally, we suggest that analysis of fat fraction distribution should account for muscle retraction and align the measurements relative to muscle length rather than position with respect to the Y-slice to allow for better comparison among datasets.

Discrepancies between the slice-wise fat fraction analysis on sagittal and coronal MRI at the same positions observed in this study can be primarily attributed to the differently oriented low-resolution direction of the MRI, resulting in different fat fraction interpolation outcomes and imperfectly aligned rotator cuff masks generated on sagittal and coronal MRI. Rather than transferring the quantitative fat fraction from the isotropic 2pDixon MRI to the lower-resolution T1-weighted MRI, we suggest that, in future studies, both the sagittal and coronal MRI, along with the rotator cuff masks, could be interpolated to an isotropic fat fraction mask for slice-wise fat fraction evaluation.

Our fat fraction prediction network was trained and tested on coronal and sagittal T1-weighted MRI and 2pDixon MRI from two centres. The Network’s generalizability to different scanner vendors, sequences or acquisition parameters (TR/TE/BW) remains untested and its performance on data from other centres need to be evaluated. In the future, the method will be tested and retrained on a large multi-centre dataset. Furthermore, it could be extended to allow for the prediction of quantitative fat fraction from other MRI modalities, however, the potential accuracy loss when applying the algorithm to other MRI modalities (e.g. PD- or T2-weighted MRI) would need to be assessed.

**Fig. 8:**
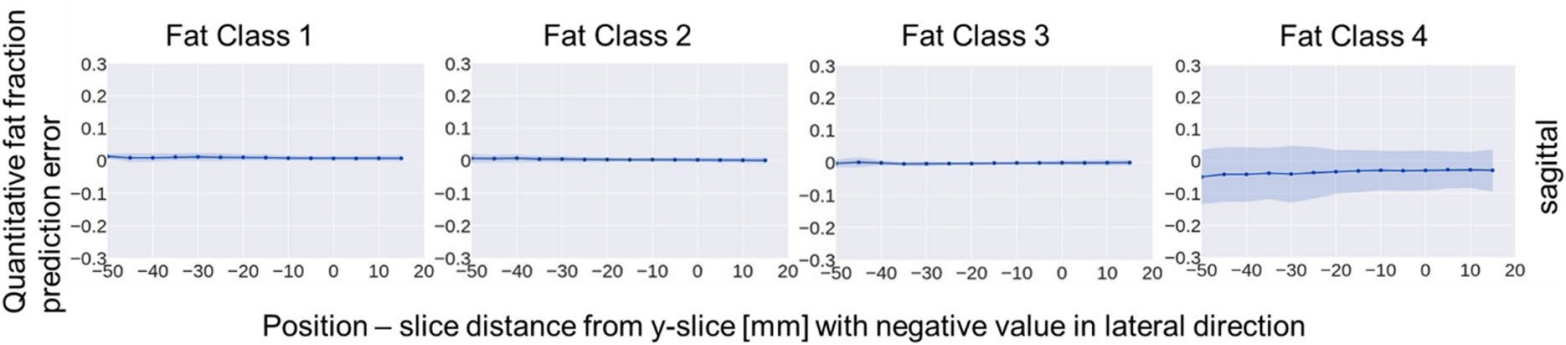
Mean and standard deviation of the errors of the predicted slice-wise quantitative fat fraction error for each of the four fat fraction classes of the test dataset compared to the corresponding ground truth for the sagittal T1-weighted MRI.

An analysis of prediction performance across fat fraction classes (see Fig. 8) indicates that while the model is highly stable for Classes 1 through 3, Class 4 (the highest fat fraction) exhibits slightly higher variance. This suggests that the algorithm effectively captures the broad muscle-fat distribution, while extreme fatty infiltration representing an area for targeted precision refinement. Future work will explore whether moving from discrete classification to continuous regression, or implementing class-specific loss weighting, can further refine the accuracy of these high-fat regions.

By enabling quantitative fat fraction analysis on T1-weighted MRI acquired during clinical routine, the presented algorithm can be widely applied and allows for higher accuracy fat fraction calculation across the entire muscle. Automation of the methods also allows for consistency across large datasets. The prognostic value of the proposed 3D measurement will be evaluated in a multicentre study investigating factors influencing the outcome of arthroscopic RC repair^36^. By enabling standardised automatic fat fraction analysis in various muscle regions, this work also has the potential to allow for a more comprehensive analysis of the RC muscles’ quality, which may help to improve the predictability of surgical outcomes.

## Data Availability

The data that support the findings of this study are available on request from K.G. The data is not publicly available due to the contained information that could compromise the privacy of the research participants.
